# A novel copper metabolism-related signature model for predicting the prognosis, target drugs, and immunotherapy in stomach adenocarcinoma

**DOI:** 10.1016/j.gendis.2023.101102

**Published:** 2023-09-14

**Authors:** Kai Zhuang, Siqi Tang, Haixin Feng, Jinying Zhang, Ying Liu, Yong Liu, Yongjian Su, Jiaqi Yu, Zunnan Huang

**Affiliations:** a Key Laboratory of Computer-Aided Drug Design of Dongguan City, The First Dongguan Affiliated Hospital, Guangdong Medical University, Dongguan, Guangdong 523710, China; b Key Laboratory of Big Data Mining and Precision Drug Design of Guangdong Medical University, Key Laboratory for Research and Development of Natural Drugs of Guangdong Province, School of Pharmacy, Guangdong Medical University, Dongguan, Guangdong 523808, China; c School of Public Health, Guangdong Medical University, Dongguan, Guangdong 523808, China; d Marine Biomedical Research Institute of Guangdong Zhanjiang, Zhanjiang, Guangdong 524203, China

Stomach adenocarcinoma (STAD) is one of the most common gastric neoplasms with a high death rate. Therefore, there is an urgent need to propose an efficient therapy for STAD. Copper plays key roles in regulating the distribution of immune cells and affecting the tumor immune escape, and may be a novel indicator of immunotherapy in STAD. However, the specific impact of copper metabolism-related genes (CMRGs) on the patient's prognosis, tumor microenvironment, and immunotherapeutic response remains unelucidated.

In this study, differential expression analysis and multivariate Cox regression analysis were conducted to construct a copper metabolism-related prognostic signature (CMRPS) model consisting of 11 prognostic signature genes. A survival plot and a receiver operating characteristic (ROC) plot were drawn to illuminate the reliability and sensitivity of this model with the area under the ROC curve of 5-year survival time (AUC_5–year_). The biological functions of the CMRPS were further explored by gene set variation analysis (GSVA), and the results indicated that copper metabolism might affect the immune pathways and metabolic reprogramming. Additionally, we performed multiple immunotherapy-related analyses and revealed that comparing two risk groups, the low-risk group responded better to immunotherapy, while the high-risk group manifested a higher drug sensitivity to common anticancer drugs. The flow diagram of this study is shown in Figure 1.

**Figure 1:**
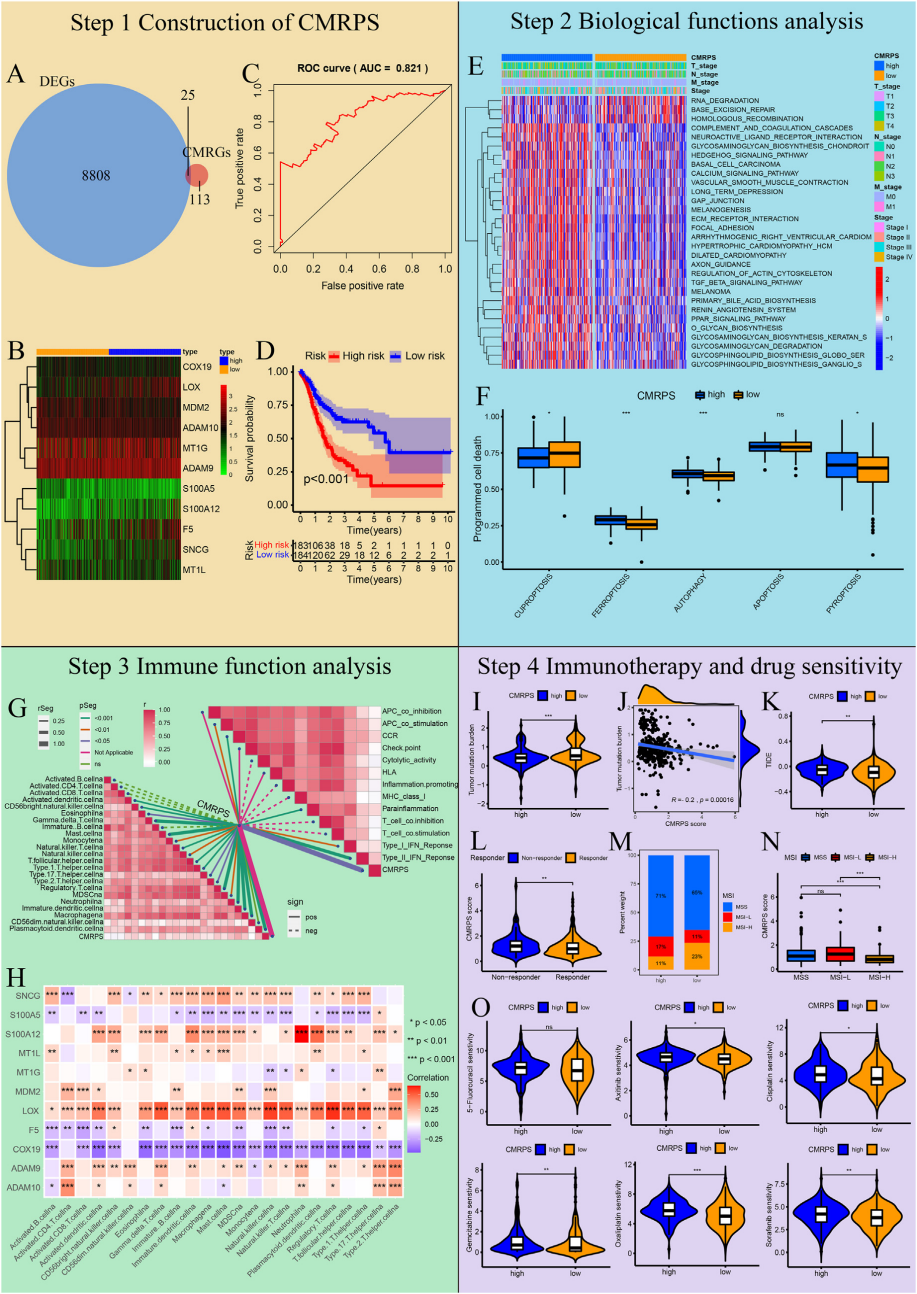
Construction of CMRPS in STAD. **(A)** Venn plot was used to identify DEGs_-CM_ between DEGs and CMRGs. The overlapping region represents the shared components between the two sets. **(B)** Heatmap of 11 DEGs-_CMRPS_ in CMRPS, comparing their expression levels between the high-risk and low-risk groups. The heatmap depicts gene expression levels in individual samples, where blue denotes the high-risk group, and orange denotes the low-risk group. Each square represents the expression level of a particular gene in a specific sample, with red indicating high expression and green indicating low expression. **(C)** ROC curve and its AUC value plot for 5-year survival time. A higher AUC value indicates a greater accuracy of the model. **(D)** Survival plot. The red curve indicates the high-risk group, and the blue curve indicates the low-risk group. *P* < 0.05 denotes statistical significance. **(E)** Heatmap of top 30 KEGG pathways in two groups. The higher levels of KEGG pathways are represented by red, whereas the lower levels are represented by blue. **(F)** ssGSEA analysis of programmed cell death in two groups. The abscissa denotes the gene sets of five forms of programmed cell death, and the ordinate denotes the enrichment scores of gene sets in the programmed cell death pathways. **(G)** Correlations plot of the CMRPS model and tumor immune microenvironment. A darker shade of red indicates a higher correlation coefficient. The solid lines denote positive correlations, and the dashed lines indicate negative correlations. **(H)** Correlation plot between the expression of 11 DEGs_-CMRPS_ and the infiltration of 23 types of immune cells. The red indicates a positive correlation, and the blue denotes a negative correlation. **(I)** Comparison of tumor mutation burden in two risk groups. **(J)** Correlation analysis of CMRPS score and tumor mutation burden. **(K)** Comparison of TIDE score in two risk groups. **(L)** Comparison of CMRPS score in two immunotherapy response groups. **(M)** Distribution of MSI in the high- and low-risk groups. **(N)** Comparison of CMRPS score in three MSI subtypes. **(O)** Sensitivity comparison of six common anticancer drugs (5-fluorouracil, axitinib, cisplatin, oxaliplatin, gemcitabine, and sorafenid) in two risk groups. The abscissa denotes the risk groups. The ordinate denotes the half-maximal inhibitory concentration (IC_50_) of the drug. The higher the IC_50_, the less the sensitivity of the cancer cells to the drug. ∗*P* < 0.05, ∗∗*P* < 0.01, ∗∗∗*P* < 0.001; ns, not significant.

First, the expression profile and the clinical data of 375 STAD samples and 32 normal samples were downloaded from The Cancer Genome Atlas (TCGA) database, and 8833 differentially expressed genes (DEGs) in STAD samples compared with normal samples were identified through the differential expression analysis using the Wilcoxon rank-sum test in R software (Fig. S1A). Moreover, we extracted the gene sets related to copper metabolism from the Molecular Signature Database version 7.1 (MSigDB v7.1),^1^ and obtained 138 CMRGs (Appendix A) after removing overlapping genes. Afterward, we identified 25 DEGs related to copper metabolism (DEGs-_CM_) from the intersection between 8833 DEGs and 138 CMRGs in STAD (Fig. 1A; Fig. S1B).

By the multivariate Cox regression analysis of 25 DEGs_-CM_, we further constructed a prognostic CMRPS model, which was composed of 11 DEGs in CMRPS (DEGs_-CMRPS_) (Fig. 1B and Table S1). Then, we computed the CMRPS score of every patient through the Cox regression equation of the CMRPS model and separated the patients into high- and low-risk clusters according to their median values (Fig. S1C, D). The reliability of the CMRPS model was tested by the ROC curve where AUC_5–year_ was 0.821 (Fig. 1C). The Kaplan–Meier survival curve of the two groups suggested that the 1- to 10-year patient survival rates in the low-risk group surpassed those in the high-risk group (Fig. 1D).

Second, GSVA was then used to reveal potential biological functional differences between two risk groups of the CMRPS model. Taking the expression levels of KEGG pathway gene set in “seth.all.v7.1.symbols” as inputs^1^ and *P* < 0.05 as the significance criterion, 59 KEGG pathways were found to be enriched in two risk groups, and the enrichment calculations were conducted using the “GSVA” package in R. As shown in Fig. 1E and Appendix B, the high-risk group was enriched in 45 pathways, including four immune pathways (complement and coagulation cascades pathway, cytokine–cytokine receptor interaction pathway, TGF-β signaling pathway, and leukocyte transendothelial migration pathway) and three metabolic pathways (arachidonic acid metabolism pathway, drug metabolism cytochrome P450 pathway, and taurine and hypotaurine metabolism pathway), while the low-risk group was enriched in 14 pathways, including another three metabolic pathways (sulfur metabolism pathway, metabolism of xenobiotics by cytochrome P450 pathway, and glyoxylate and dicarboxylate metabolism pathway), indicating the significant disparities in immune- and metabolic-related pathways between two CMRPS groups.

Subsequently, single-sample gene set enrichment analysis (ssGSEA)^2^ was performed based on the expression levels of five gene sets (Appendix C) classified in programmed cell death pathways. It was observed with statistical significance that the enrichment score of cuproptosis-related genes in the low-risk group was higher than that in the high-risk group, the direct opposite trend to that of ferroptosis-, autophagy- and pyroptosis-related genes (all *P* < 0.05), suggesting the impact of copper metabolism on programmed cell death (Fig. 1F).

Third, we also used ssGSEA to investigate the potential connections between the CMRPS model and the tumor immune microenvironment. According to the expression levels of 38 reported immune-related gene sets, which were classified into 23 immune cell infiltrations (Appendix D)^3^ and 15 immune functions (Appendix E),^4^ positive correlations with statistical significance (*P* < 0.05) were obtained between the CMRPS scores and the infiltration degrees of 18 immune cells (Activated.dendritic.cellna, CD56bright.natural.killer.cellna, Eosinophilna, and Gamma.delta.T.cellna, *etc*.) and between the CMRPS scores and the function scores of five immune bioprocesses (APC- co_stimulation, CCR, Parainflammation, Type I IFN Response, and Type II IFN Response) (Fig. 1G). Furthermore, the Spearman correlations between 23 immune cell infiltrations and 11 DEGs-_CMRPS_ showed that *S100A12*, *MDM2*, *LOX*, and *ADAM9* were immune genes while S100A5, *F5*, and *COX19* were anti-immune genes, as their expression were positively (former) or negatively (latter) associated with the infiltration degrees of immune cells in STAD samples (Fig. 1H). The findings highlighted the significant role of copper metabolism in the tumor immune microenvironment.

Fourth, we explored the relationship of CMRPS with the immunotherapy response. By comparing the somatic mutation data of patients in two CMRPS groups based on single nucleotide variant analysis using the “maftools” package in R, we observed that the low-risk group held a higher tumor mutation burden than the high-risk group (*P* < 0.001; Fig. 1I; Fig. S2A, B). The CMRPS scores showed a negative association with the tumor mutation burden of patients with STAD (*r* = −0.2, *P* = 0.00016; Fig. 1J). In addition, tumor immune dysfunction and exclusion (TIDE) analysis based on mRNA expression in patients with STAD indicated a lower TIDE score in the low-risk group compared with the high-risk group (*P* < 0.01; Fig. 1K), and the responder group had a lower CMRPS score than the non-responder group (*P* < 0.01; Fig. 1L). Furthermore, by comparing the composition of patients' microsatellite instability (MSI) subtypes from The Cancer Immunome Atlas (TCIA) website^3^ between two CMRPS groups, we observed that the low-risk group possessed a higher proportion of MSI-H (23%) than the high-risk group (11%), suggesting a relatively higher immunotherapy sensitivity of patients in the low-risk group (Fig. 1M). The CMRPS scores of patients with STAD were statistically different between patients with MSS and MSI-H and between patients with MSI-L and MSI-H (*P* < 0.001; Fig. 1N). This shows that the CMRPS score of the CMRPS model might help the sectionalization of MSI and the choice of the immunotherapy type. Thus, the correlation between the CMRPS and immunotherapy response provided insights into the potential utility of the CMRPS model in guiding personalized immunotherapy strategies.

Finally, we evaluated the therapeutic drug reaction of two CMRPS groups to six common anticancer drugs using the “oncoPredict” package in R according to drug sensitivity data, the half maximum inhibition concentration (IC50), from the Genomics of Drug Sensitivity in Cancer database (GDSC)^5^ and mRNA expressions of patients with STAD. The low-risk group was observed to be more sensitive to five of these drugs (axitinib, cisplatin, gemcitabine, oxaliplatin, and sorafenib) but not 5-fluorouracil than the high-risk group with statistical significance (Fig. 1O), denoting that the CMRPS model could serve as a reliable indicator of drug sensitivity to facilitate accurate, personalized treatment for patients with cancer.

In this study, we established a copper metabolism-related prognostic signature that functioned as the predictor of patient prognosis and further revealed that copper metabolism might have important roles in the immune-related pathways and metabolic reprogramming-related pathways in STAD. In addition, copper metabolism could affect programmed cell death and sensitivity to anticancer drugs. In summary, our study has contributed to the understanding of the correlations among copper metabolism, the tumor microenvironment, and immunotherapy in STAD and provided new prognostic biomarkers, therapeutic targets, and immunotherapeutic indicators for the clinical research and treatment of STAD.

## Author contributions

Kai Zhuang: conceptualization, data curation, formal analysis; Siqi Tang: writing - review & editing; Haixin Feng: formal analysis, writing - original draft; Jinying Zhang: writing - original draft; Ying Liu: formal analysis; Yong Liu: writing - review & editing; Yongjian Su: writing - review & editing; Jiaqi Yu: visualization; Zunnan Huang: conceptualization, writing - review & editing, supervision, funding acquisition.

## Conflict of interests

The authors declare that they have no competing interests.

## Funding

This work was supported by the Key Discipline Construction Project of Guangdong Medical University (No. 4SG22004G), the Higher Education Reform Project of Guangdong Province, China (No. 2019268), the “Climbing Project” Special Fund for Science and Technology Innovation Cultivation of College Students in Guangdong Province, China (No. pdjh2021b0225, pdjh2022a0217), and the Innovation and Entrepreneurship Training Program for students of Guangdong Medical University (No. 202110571013).

## Data availability

The datasets analyzed during the current study are publicly available in the TCGA at https://portal.gdc.cancer.gov/.

## References

[1] Liberzon A., Subramanian A., Pinchback R., Thorvaldsdóttir H., Tamayo P., Mesirov J.P. (2011). Molecular signatures database (MSigDB) 3.0. Bioinformatics.

[2] Barbie D.A., Tamayo P., Boehm J.S. (2009). Systematic RNA interference reveals that oncogenic KRAS-driven cancers require TBK1. Nature.

[3] Charoentong P., Finotello F., Angelova M. (2017). Pan-cancer immunogenomic analyses reveal genotype-immunophenotype relationships and predictors of response to checkpoint blockade. Cell Rep.

[4] He Y., Jiang Z., Chen C., Wang X. (2018). Classification of triple-negative breast cancers based on immunogenomic profiling. J Exp Clin Cancer Res.

[5] Yang W., Soares J., Greninger P. (2013). Genomics of Drug Sensitivity in Cancer (GDSC): a resource for therapeutic biomarker discovery in cancer cells. Nucleic Acids Res.

