# Supplementary material for: A novel copper metabolism-related signature model for predicting the prognosis, target drugs, and immunotherapy in stomach adenocarcinoma
Source: Genes Dis. 2023 Sep 14;11(5):101102. doi: 10.1016/j.gendis.2023.101102 (PMC11106529; doi:10.1016/j.gendis.2023.101102)
Supplement: Multimedia component 1 [file mmc1.docx]

**Supplementary Document**

**A novel copper metabolism-related signature model predicting the prognosis,** **target drugs, and immunotherapy in** **stomach adenocarcinoma**

Kai Zhuang, Siqi Tang, Haixin Feng, Jinying Zhang, Ying Liu, Yong Liu, Yongjian Su, Jiaqi Yu, Zunnan Huang

**Introduction**

Stomach adenocarcinoma (STAD) is one of the most common gastric malignancies with a poor prognosis. According to the global epidemiological investigation in 2020, STAD ranked fifth in cancer morbidity and fourth in cancer-related mortality.^1^ The oncogenesis and progression mechanism of STAD remains largely unilluminated, and the biomarkers clinically used today have underperformed in predicting the prognosis in patients with STAD. The precise diagnosis and treatment of STAD is an urgent public health problem, which calls for further research on the mechanism of STAD. The discovery and application of novel prognostic biomarkers could improve the accuracy of prognostic prediction and the effect of clinical treatment in patients with STAD.

Copper metabolism is a novel antitumor direction frequently highlighted in recent articles.^2−4^ It was reported that abnormally copper metabolism could promote a mitochondrial metabolic reprogramming in the reformation of the oxidative phosphorylation system to increase the production of reactive oxygen species (ROS), decrease the mitochondrial ATP level, and induce metastasis and drug-resistance of tumor cells.^4−6^ Copper ion is indispensable for cuproptosis, a type of programmed cell death.^7^ When the accumulation of copper ions reaches a certain level in cells, it will become cytotoxic and lead to copper-induced cell death. Moreover, copper ion plays essential roles in various programmed cell death mechanisms, and the concentration of copper in cells directly affects the sensitivity of cells to death.^8−11^ However, there are limited studies regarding copper metabolism and the function of copper metabolism-related genes (CMRGs) in STAD. Deeply exploring the relationships between copper metabolism and STAD may provide an opportunity to develop a new model that recognizes and characterizes copper-dependent signals and to find a new direction for the clinical therapy of STAD.

The development of immune therapy for tumors is gradually challenging traditional treatments, including chemotherapy and radiation therapy, and it benefits patients with STAD with a better prognosis.^12^ Many inhibitors of immune checkpoints are proposed to improve the survival rate and suggested to be potential novel candidate treatments for patients with STAD.^13−15^ However, positive reactions were only observed in a few patients with STAD.^13−15^ It is urgently needed to find new predictive signatures for immune therapy for STAD to determine the applicable population. In addition, it is widely reported that copper metabolism could affect the distribution and the number of immune cells by impacting mitochondrial metabolic reprogramming and might influence the immunosuppression of immune cells and the immune microenvironment.^6,16^ A plethora of research has been conducted to reveal the characteristic biomarkers for immune therapy in STAD, and these signatures were helpful in determining the population of patients with STAD applicable for immunotherapy.^17−19^ Nevertheless, the detailed immune functions of CMRGs in STAD remain to be systematically investigated.

Based on the STAD dataset derived from The Cancer Genome Atlas (TCGA) database, we explored the mechanism of copper metabolism in the immune microenvironment, immunotherapy, and precision therapy of STAD from the perspectives of gene expression, prognostic model, immune cell infiltration, biological pathways, gene mutation, and target drugs. The novel results revealed by this study will contribute to the immune treatment selection for patients with STAD in clinical work, as well as provide logical foundations for the regulation of copper metabolism and the prediction of the immunotherapeutic response in patients with STAD.

**Materials and methods**

***Data preparation***

The expression profile and the clinical data of 375 STAD samples and 32 normal samples were obtained from TCGA database (https://portal.gdc.cancer.gov/) on March 20, 2022. The expression profile was converted into fragments per kilobase of exon model per million mapped fragments (FPKM), which increased the comparability of gene expression between samples.

Moreover, we extracted the data of gene sets related to copper metabolism from the Molecular Signature Database version 7.1 (MSigDB v7.1)^20,21^ and obtained CMRGs after removing overlapping genes.

***Differential expression analysis***

The Wilcoxon rank-sum test and "limma" package in R software were used to analyze the gene expression profile of STAD samples for identifying the differentially expressed genes (DEGs) between STAD samples and normal gastric samples. Afterward, the DEGs and CMRGs were intersected to obtain the differentially expressed genes related to copper metabolism (DEGs-_CM_), and the results were visualized as the heatmap and Venn diagram of differential expression. The cutoff criteria were logarithmic fold-change (logFC) of 1 and false discovery rate (FDR) of 0.05.

***Establishment of copper metabolism-related prognostic signature (CMRPS)***

Multivariate Cox proportional risk regression analysis was performed on DEGs-_CM_ to construct a CMRPS model and evaluate the prognostic effect of the prognostic DEGs-_CM_ in the CMRPS model (DEGs-_CMRPS_) on patients' survival time. According to the Cox proportional risk regression model, the CMRPS score of each patient was calculated by the following formula:

CMRPS score = [(Exp_gene1_ × *β*_gene1_) + (Exp_gene2_ × *β*_gene2_) +(Exp_gene3_ × *β*_gene3_)] + ... + (Exp_gene_*_n_* ×*β*_gene_*_n_*).

Subsequently, according to the median value of the CMRPS score, patients with STAD were separated into high-risk and low-risk groups. CMRPS score curves and survival status maps were drawn to show the difference in CMRPS score and survival status between each sample. A Kaplan−Meier curve was constructed to compare the differences in survival trends between the two risk groups with *P* < 0.05 as the cutoff standard, and the logarithmic rank test was conducted to evaluate their discrepancy in survival time. The "timeROC" package in R software was used for time-dependent receiver operating characteristic (ROC) curve analysis, and the area under the ROC curve of 5-year survival time (AUC-5 year) between the two risk groups was calculated to evaluate the sensitivity and reliability of the CMRPS model.

***Analysis of biological functional pathway***

Gene set variation analysis (GSVA) could enrich the biological functions of gene sets in specific samples. Using the hallmark gene set "h.all.v7.1.symbols" in the MSigDB v7.1 as the reference gene set,^20^ GSVA enrichment scores of the Kyoto Encyclopedia of Genes and Genomes (KEGG) pathways were separately identified according to the expression levels of set genes for the high-risk group and the low-risk group by using the "GSVA" package in R software. *P* < 0.05 was set as the criterion of statistical significance.

Furthermore, the effect of copper metabolism on five forms of programmed cell death (cuproptosis, ferroptosis, autophagy, apoptosis, and pyroptosis) in the high-risk and low-risk groups was explored by single-sample gene set enrichment analysis (ssGSEA) by using the "GSVA" package. Among them, cuproptosis-related genes were extracted from the literature,^7^ 264 ferroptosis driver genes were downloaded from the FerrDb website (http://www.zhounan.org/ferrdb),^22^ and autophagy-, apoptosis-, and pyroptosis-related genes were extracted from MSigDB v7.1. We calculated the enrichment score of each sample in the high- and low-risk groups according to the expression levels of the above five gene sets and compared the scores of the five forms in the programmed cell death pathway between the two risk groups. *P* < 0.05 was set as the criterion of statistical significance.

***Immune function analysis***

To investigate the level of the tumor immune microenvironment in each CMRPS patient, we collected 23 immune cell infiltration gene sets and 15 immune function gene sets from previous reports.^23,24^ The ssGSEA method was then used to assess the scores of these immune cell infiltrations or immune functions of all patients in the CMRPS model, and the Pearson correlation analysis was performed to construct the potential connections between these immune scores and their CMRPS scores. In addition, the Spearman correlations between 11 DEGs_-CMRPS_ and 23 immune cell infiltrations were also explored to evaluate the potential effect of these copper metabolism-related prognostic genes on the STAD immune microenvironment. *P* < 0.05 was set as the criterion of statistical significance.

***Somatic mutation analysis (SNV)***

To evaluate the somatic mutation degree of CMRPS in the high-risk and low-risk groups, we collected the somatic mutation data of STAD samples from the XENA database (<https://xenabrowser.net>)^25^ and performed SNV by using the "maftools" package^26^ in R software.

Tumor mutation burden (TMB) is the sum of somatic gene insertions or deletions, gene coding errors, and base substitutions detected per million bases of patients,^27,28^ which was also calculated by using the "maftools" package. Then, we conducted a Spearman correlation analysis to investigate the relationships between the TMB and the CMRPS scores of patients. Meanwhile, violin plots were constructed to compare the TMB of different CMRPS groups. *P* < 0.05 was set as the cutoff criterion.

***Therapeutic efficacy of immunotherapy***

Three analyses, including tumor immune dysfunction and exclusion (TIDE), immunophenoscore (IPS), and microsatellite instability (MSI), were employed to test the therapeutic efficacy of the CMRPS model in immunotherapy.

The TIDE web tool (http://tide.dfci.harvard.edu/)^29^ was used to predict the immunotherapy response of CMRPS patients to immune checkpoint blockade (ICB) and evaluate their TIDE scores to provide a more accurate assessment of immune escape levels based on mRNA expression in patients with STAD. Violin plots were generated to compare the TIDE scores of different CMRPS groups and the CMRPS scores of different response groups to immunotherapy. These plots were used to estimate the relationship between the CMRPS scores and the efficacy of immunotherapy.

The Cancer Immunome Atlas (TCIA, [https://www.tcia.at/home)](https://www.tcia.at/home).) is an integrated repository of immune-related data for 20 cancer types derived from the TCGA project.^23^ It encompasses valuable information on patients' MSI and IPS. MSI is a molecular character of a dysfunctional mismatch repair system and a marker of cell susceptibility to immunotherapy.^30^ It includes MSI-high (MSI-H), MSI-low (MSI-L), and MS-stable (MSS). Information on the patient's MSI subtype was also directly obtained from TCIA[.](https://www.tcia.at/home).) The MSI subtypes composition in the high-risk and low-risk groups was percentage-wise analyzed and visualized as a histogram. Violin plots were drawn to compare the differences in CMRPS scores across MSI subtypes. The IPS is a novel indicator that predicts the response to ICB therapy.^23^ We constructed violin plots comparing the IPS of immunotherapy response to the single use of cytotoxic T-lymphocyte–associated antigen 4 (CTLA-4), programmed death 1 (PD-1), or the combined use of CTLA-4 and PD-1 in the two risk groups of the CMRPS model. *P* < 0.05 was set as the criterion of statistical significance.

***Therapeutic drug analysis***

To evaluate therapeutic drug reaction in CMRPS, we selected six classical anticancer drugs included in the Genomics of Drug Sensitivity in Cancer (GDSC) database (https://www.cancerrxgene.org/)^31^ from a review paper authored by Wu^32^. The "oncoPredict" package in R software was then performed to forecast the anticancer drug sensitivity according to mRNA expressions of patients with STAD using a ridge regression model, which was constructed in priority based on a training set including both the expression data of genes in tumor cells and the half-maximal inhibitory concentration (IC_50_) data of the drugs from the GDSC database. In addition, the CellMiner database (<https://discover.nci.nih.gov/cellminer/home.do>)^33,34^ was used to identify candidate drugs targeting DEGs-_CMRPS_ in patients with STAD according to tumor cell gene expressions and drug IC_50_ data stored in-house. Compounds with correlation coefficients (*r*) greater than 0.4 and *P* < 0.001 were considered as potential therapeutic drugs targeted to copper metabolism.

***Statistical analysis***

The statistical calculations in this study were performed using R 4.1.2. Group comparisons were evaluated by an independent *t*-test for continuous data and the χ^2^ test for categorical data. In the two-tailed test, *P* < 0.05 was set as the criterion of statistical significance by default.

**Result**

***Flowchart of the data mining path to investigate the functions of copper metabolism in STAD***

**Fig. 1** shows the data mining flowchart of copper metabolism in STAD. First, we downloaded the expression profile and clinical data of STAD samples from the TCGA database and performed differential expression analysis to identify DEGs. Then, CMRGs were screened out from MSigDB v7.1 and intersected with DEGs to obtain DEGs-_CM_. Afterward, through the multivariate Cox regression analysis on DEGs-_CM_, we constructed a CMRPS model which divided STAD samples into high- and low-risk groups according to the median CMRPS score. GSVA, ssGSEA, and immune function analysis were performed to enrich the KEGG pathways and compare the degree of programmed cell death and the infiltration of immune cells in two risk groups. SNV analysis was undertaken to reveal the mutation for two CMRPS groups in STAD. The MSI, TIDE, and IPS analyses were conducted to predict the sensitivity of tumor cells to immunotherapy. Finally, we compared the drug sensitivity of common anticancer drugs and identified candidate drugs targeting the DEGs-_CMRPS_ from the CellMiner database.

***DEGs, CMRGs, and DEGs-_CM_ in STAD***

Through the differential expression analysis of 375 STAD and 32 normal samples, we obtained 8,833 DEGs. **Fig. S1A** shows the volcano plot of DEGs. It manifested the significantly abnormal expression levels of DEGs in STAD compared to normal gastric samples. Among the 8,833 DEGs, 7,498 genes were upregulated, and 1,335 genes were downregulated.

After a systematic search in MSigDB v7.1, 138 CMRGs were extracted after removing duplicate genes (**Appendix A**). Then, we intersected 8,833 DEGs and 138 CMRGs and identified 25 DEGs-_CM_ (**Fig. 1A**), including 13 overexpressed and 12 underexpressed genes (**Fig. S1B**).

***CMRPS prediction model in STAD***

According to 25 DEGs-_CM_, a prognostic CMRPS model was constructed by the multivariate Cox regression analysis, which included 11 DEGs-_CMRPS_: *LOX*, *F5*, *S100A5*, *ADAM9*, *ADAM10*, *COX19*, *MDM2*, *MT1L*, *SNCG*, *S100A12*, and *MT1G* (**Fig. 1B** and **Table S1**).

Next, the CMRPS model was constructed to determine the CMRPS score for each patient. Based on the median value of the CMRPS score, the patients were categorized into either the high-risk or low-risk group (**Fig. S1C** and **D**). The ROC curve showed a 5-year AUC value of 0.821, indicating that this CMRPS model has high predictive power (**Fig. 1C**). The Kaplan−Meier survival curve revealed that patients in the low-risk group exhibited higher survival rates over a 10-year period compared to those in the high-risk group (**Fig. 1D**).

***Biological pathways enriched in*** ***two risk groups***

The GSEA showed that a total of 59 KEGG pathways were enriched in the two risk groups of the CMRPS model (**Fig. 1E** and **Appendix B**), among which 45 pathways (including four immune pathways and three metabolic pathways) were upregulated in the high-risk group, and the remaining 14 pathways (including another three metabolic pathways) were upregulated in the low-risk group, as described in more detail in the letter to the editor.

Moreover, the ssGSEA of five gene sets (**Appendix C**) involved in programmed cell death displayed that the low-risk group held higher enrichment scores for cuproptosis-related genes compared to the high-risk group (*P* < 0.05), and the enrichment scores of ferroptosis- (*P* < 0.001), autophagy- (*P* < 0.001), and pyroptosis-related genes (*P* < 0.05) in the high-risk group were higher than the corresponding ones in the low-risk group (**Fig. 1F**).

***Immune infiltration analysis of CMRPS***

Additional ssGSEA using the expression levels of 23 immune cell infiltration gene sets (**Appendix D**) and 15 immune function gene sets (**Appendix E**) showed the connection between the CMRPS model and the immune cells or bioprocesses (**Fig. 1G**). The graph in the lower right corner of **Fig. 1G** indicates positive correlations between the CMRPS scores calculated based on the CMRPS model and the infiltrations of 18 immune cell types (*P* < 0.05), while the graph in the higher left corner of **Fig. 1G** denotes positive connections between the CMRPS scores of the CMRPS model and the function scores of five immune bioprocess types (*P* < 0.05). Furthermore, **Fig. 1F** shows the Spearman correlation between the infiltration degrees of 23 immune cells and the expression levels of 11 DEGs-_CMRPS_, which were described more in the letter to the editor.

***Mutation landscape of*** ***DEGs-_CMRPS_ and TMB analysis of CMRPS***

We quantified the mutation percentages in two risk groups and visualized them as waterfall plots (**Fig. S2A** and **B**). The top five genes with the highest mutation frequency in the high-risk group were *TTN* (52%), *TP53* (41%), *MUC16* (36%), *SYNE1* (31%), and *CSMD3* (29%), and the top five genes with the highest mutation frequency in the low-risk group were *TTN* (64%), *TP53* (41%), *MUC16* (37%), *LRP1B* (31%), and *SYNE1* (31%). The overall mutation rate in the low-risk group was relatively higher than in the high-risk group (91.21% versus 86.81%).

The TMB analysis showed that the TMB of the high-risk group was lower than that of the low-risk group (**Fig. 1I**), as a significant and negative correlation existed between the CMRPS scores and TMB of STAD (*P* = 0.00016, **Fig. 1J**).

***Immunotherapy response of CMRPS***

The TIDE analysis revealed that the low-risk group had a lower TIDE score than the high-risk group (*P* < 0.01, **Fig. 1K**). In addition, we compared the differences in CMRPS scores between the TIDE-predicted immunotherapy response groups. The result manifested that the responder group had a lower CMRPS score than the non-responder group (*P* < 0.01, **Fig. 1L**).

The MSI analysis showed that the high-risk group was composed of 71% MSS, 17% MSI-L, and 11% MSI-H, and the low-risk group consisted of 65% MSS, 11% MSI-L, and 23% MSI-H (**Fig. 1M**). The CMRPS scores of patients with STAD were statistically different between patients with MSS and with MSI-H (*P* < 0.001) and between patients with MSI-L and with MSI-H (*P* < 0.001), but not between patients with MSS and MSI-L (*P* > 0.05) (**Fig. 1N**).

Next, we predicted the immunotherapeutic response of STAD samples to CTLA4 or PD-L1 or their combination. The analysis results manifested that the IPS of the high- and low-risk groups had no significant difference without CTLA-4 or PD-1 treatment (**Fig. S2C**), whereas the IPS of the low-risk group was higher than that of the high-risk group under the respective or the combined treatment of CTLA-4 and PD-1 (*P* < 0.05, **Fig. S2D−F**). Thus, these findings demonstrated that the low-risk group displayed a more favorable immunotherapy response, and the CMRPS score could assist in the prediction of the immunotherapy sensitivity of patients with STAD.

***Drug sensitivity of CMRPS***

The "OncoPredict" analysis based on GDSC data revealed that the patients in the low-risk group were more sensitive to five of six common anticancer drugs, including axitinib (*P* < 0.05), cisplatin (*P* < 0.05), gemcitabine (*P* < 0.001), oxaliplatin (*P* < 0.01), and sorafenib (*P* < 0.01), but not 5-fluorouracil (*P* > 0.05) than the patients in the high-risk group (**Fig. 1O**).

CellMiner analysis based on the correlation between the drug IC_50_ and the expression levels of 11 DEGs-_CMRPS_ identified 141 pairs of candidate drugs and their target genes (**Appendix F**). The top nine drug-gene pairs were taken as examples, and their correlations were visualized as scatter plots (**Fig. S2G**). Among the nine candidate drugs, six drugs had IC_50_ values associated with the expression level of *S100A12* (*P* < 0.001): GSK-2194069 (*r* = 0.641), ABT-199 (*r* = 0.636), imexon (*r* = 0.634), cyclophosphamide (*r* = 0.600), and nandrolone phenpropionate (*r* = 0.575) manifested positive connections, and INK-128 (*r* = −0.646) presented a negative correlation. In addition, three other drugs formed the remaining pairs with three other genes, and these three pairs, including GSK-1904529A and *F5* (*r* = 0.606), P-529 and *LOX* (*r* = 0.604), and AMG-232 and *MDM2* (*r* = 0.585), showed positive associations between the drug IC_50_ value and gene expression (*P* < 0.001).

**Discussion**

STAD is one of the most common gastric malignancies with a metastasis propensity and poor prognosis. In 2020, there were 1,089,103 individuals newly diagnosed with gastric cancer, and gastric cancer caused 768,793 deaths,^1^ incurring a heavy burden on global health care. Although research on gastric cancer remains active, the mechanism of STAD has not been fully elucidated, and the therapy strategies available barely satisfy the patient's expectation of prognosis.

Recently, therapies that exploit copper metabolism or the host immune system to attack tumor cells have proposed a new direction in medical oncology. Copper metabolism disorder, which could lead to copper death in various cells, was found to exist widely in patients with tumors, especially malignant tumors, including STAD.^35−37^ Copper metabolism was also found to inhibit the function of key immune cells, such as macrophages, T helper cells, B cells, and neutrophil natural killer (NK) cells, which play important roles in killing infectious microorganisms, mediating immunity, and producing specific antibodies against pathogens.^38^ This suggested that the inhibition of copper metabolism can promote immune cells to improve the antitumor efficacy of immunotherapy. However, no studies have reported a direct relationship between CMRGs and immunotherapy in STAD. Therefore, further investigation of copper metabolism and immune functions in STAD may contribute to the development of prognostic markers, elucidation of oncogenesis mechanisms, exploration of tumor immunotherapy, and practice of precision therapy.

In this study, we first downloaded 375 STAD samples and 32 normal gastric samples from the TCGA database and identified 8,833 DEGs through differential analysis. Next, we extracted 138 CMRGs from the MSigDB v7.1 and intersected them with 8,833 DEGs to obtain 25 DEGs-_CM_ in STAD. Then, we performed multivariate Cox risk regression analysis on the 25 DEGs-_CM_ and constructed a CMRPS Cox model including 11 DEGs-_CMRPS_, which calculated the CMRPS score of each patient and divided all samples into a high-risk group and a low-risk group according to the median score. Through the survival analysis of these two groups, we observed a longer overall survival time in the low-risk group than in the high-risk group. Based on the ROC curve analysis, the CMRPS model was evaluated as reliable in predicting the prognosis of patients with STAD (AUC-5 year = 0.821). In addition, we also conducted GSVA and ssGSEA in the two risk groups, found 59 KEGG pathways enriched in the two groups, and observed that copper metabolism in STAD was associated with immune function, metabolic reprogramming, and programmed cell death. Finally, a comprehensive literature survey was carried out to reveal the pathophysiologic roles of the 11 DEGs-_CMRPS_ of CMRPS in STAD or other cancers.

Among the 11 DEGs-_CMRPS_, seven genes (*LOX*, *F5*, *S100A5*, *ADAM9*, *ADAM10*, *COX19*, and *MDM2*) were upregulated, and four (*MT1L*, SNCG, *S100A12*, and *MT1G*) were downregulated. After systematic research and careful reading of related articles published about the 11 genes (**Table S2**), we noticed that the aberrant expression of eight genes had been experimentally reported in gastric cancer, among which the overexpression of five genes (*LOX*, *F5*, *ADAM9*, *ADAM10*, and *MDM2*) and the downregulation of three genes (*SNCG*, *S100A12*, and *MT1G*) were all consistent with our results. In addition, the decreased expression of one gene (*MT1L*) has also been experimentally verified in other tumors. The expression of *S100A5* in cancer has not been experimentally compared with its expression in normal tissues but was found to be downregulated in the recurrent cases compared with the non-recurrent cases. The remaining gene (*COX19*) is a novel biomarker for which its mechanisms in cancer are unknown, and further experimental exploration is required.

It was proposed by previous literature that copper metabolism imbalance is tightly associated with STAD,^4,5^ but the main mechanisms of copper metabolism in STAD are still undefined. Thus, we investigated the differences in the biological functional pathways for CMRPS in order to explore the related functions of CMRGs in STAD. As a result, we found that CMRPS was associated with immune function and metabolic reprogramming (**Fig. 1E**). For the high-risk group of CMRPS, three metabolic pathways (arachidonic acid metabolism pathway, drug metabolism cytochrome P450 pathway, and taurine and hypotaurine metabolism pathway) were enriched to be reprogrammed (**Appendix B**). Xu et al. elucidated that arachidonic acid metabolism, as one of the lipid metabolism pathways, could promote the formation of M2-type tumor-associated macrophages, which aggravated tumor progression in esophageal squamous cell carcinoma.^49^ McFadyen et al. proposed that one of the cytochrome P450 isoforms, CYP1B1, is the metabolizing enzyme for anticancer drugs (5-fluorouracil, cyclophosphamide, cisplatin, doxorubicin, paclitaxel, and docetaxel), and its inhibition might be a hopeful direction for renal cancer treatment.^50^ Gu et al. reported that the taurine and hypotaurine metabolism pathway was continually disturbed during the progression of gastric carcinogenesis in mice.^51^ Furthermore, short-term studies showed that copper metabolism was involved in mitochondrial function and played an important role in the Warburg effect. Cui et al. illustrated that copper metabolism in mitochondria could affect the metabolic reprogramming in cancer cells, and the use of copper-chelating agents could inhibit tumor growth.^52^ It has also been demonstrated that copper metabolism imbalance leads to changes in lipid metabolism.^53^ In addition, the metabolic reprogramming of mitochondria could regulate the immune microenvironment of a tumor to improve the effect of tumor immunotherapy.^54−56^ It could be reasonably inferred that copper metabolism might mediate metabolic reprogramming to regulate tumor immune function and is a new tumor immune regulation target. However, there is a lack of such reports, which deserves further study.

Many studies have pointed out that copper ions are widely involved in the programmed cell death of tumors.^7−11^ We explored the relationships between copper metabolism and programmed cell death. The results showed that there was a higher level of ferroptosis, autophagy, and pyroptosis in the high-risk group and a higher level of cuproptosis in the low-risk group, which means that copper metabolism could affect multiple programmed cell death mechanisms in STAD (**Fig. 1F**). Tsvetkov et al. illustrated that the disorder of copper metabolism in mitochondria affects the occurrence of cuproptosis caused by the tricarboxylic acid (TCA) cycle.^7^ It could be speculated that copper metabolism-regulated metabolic reprogramming might affect the occurrence of a variety of programmed cell death mechanisms, which may be the future research direction of programmed cell death in STAD.

Furthermore, we explored the relationships between the CMRPS score and tumor immune microenvironment in two risk groups. It was found that the CMRPS scores were positively correlated with the infiltrations of 18 immune cells and the functions of five immune bioprocesses (**Fig. 1G**), thus indicating a higher activity of immune cells in the high-risk than low-risk group and suggesting that copper metabolism might promote the effect of immune cells and the immune system. A previous study observed that cytokines could be involved in copper metabolism mechanisms to promote carcinogenesis in immune reactions.^57^ These results suggested the value of further investigation on the relationship between copper metabolism genes and immune functions.

In addition, the correlation between the expression levels of DEGs-_CMRPS_ and the infiltration degrees of the immune cells was investigated. As a result, we found that the expression levels of four DEGs-_CMRPS_ (*S100A12*, *MDM2*, *LOX*, and *ADAM9*) were mainly positively associated with the infiltration degrees of immune cells in STAD samples, whereas three DEGs-_CMRPS_ (*S100A5*, *F5*, and *COX19*) showed mainly a negative association between their expressions and the immune infiltration degrees (**Fig. 1H**). This implied that they might be immune or anti-immune genes and are the key genes in copper metabolism function to stimulate or suppress the infiltrations of immune cells in STAD. Among the four inflammation-related copper metabolism genes, Carvalho et al. reported that *S100A12* could promote NF-κB activation, pro-inflammatory signaling, and initiation of the inflammatory response by binding to and activating the RAGE receptor and could facilitate pro-inflammatory cytokine secretion and inflammatory responses through binding to TLR-4.^58^ Zhou et al. showed that competition between *MDM2* and c-Cbl for *STAT5* binding decreased c-Cbl-mediated ubiquitination and degradation of *STAT5*, and enhanced *STAT5* stability in tumor-infiltrating CD8 T cells.^59^ Moreover, advanced tumor progression and suppressed survival and function of tumor-infiltrating CD8 T cells were observed in mice with *MDM2*-deficient T cells.^59^ Huang et al. revealed that *LOX* expression was significantly increased in cancer-associated fibroblasts, which led to the epithelial−mesenchymal transition and poor prognosis in osteosarcoma, and found that *LOX* could modulate cancer-associated fibroblast (CAF) function and macrophage polarization to reshape the tumor immune microenvironment in osteosarcoma.^60^ Umeda et al. illuminated that *ADAM9* could activate TGF-β1 to promote Th17 cell differentiation.^61^ Among the three immunosuppression-related copper metabolism genes, Wang et al. pointed out that *F5* could act as a T-cell inhibitor of leukocyte expression.^62^ *S100A5* and *COX19* are novel anti-immune targets first reported for their roles in immune function in our study. These findings suggest that copper metabolism genes might influence the immune function of STAD by regulating the copper metabolism of STAD.

Immunotherapy has been clinically used in the treatment of STAD. However, STAD immunotherapy is faced with low response rates.^13−15^ Therefore, it is urgently needed to find novel biomarkers that precisely identify suitable patients for immunotherapy. Thus, we chose immunotherapy in STAD as a focus point. SNV was conducted to clarify the mutation landscape and TMB in two risk groups of the CMRPS model. It was noted that TMB was negatively associated with the CMRPS score (**Fig. 1J**), which implied that the high-risk group had a lower mutation frequency and TMB than the low-risk group (**Fig. 1I** and **Fig. S2A** and **B**). It is commonly known that the subtype of MSI is correlated with the sensitivity of tumor cells to immunotherapy, and the TIDE score is correlated with the trend of immune escape. Tumors with the MSI-H subtype show the best response to immunotherapy among the three MSI subtypes (MSS, MSI-L, and MSI-H), and tumors with a higher TIDE score have a higher tendency of immune escape. We conducted MSI and TIDE analyses in the CMRPS groups and observed a higher proportion of MSI-H subtypes (23%) and a lower TIDE score (*P* < 0.01) in the low-risk group than in the high-risk group (11%) (**Fig. 1K** and **M**). These results suggested that the tumor cells of STAD in patients in the low-risk group may have a relatively better immunotherapy response and a lower trend of immune escape, which concurred with our outcomes that the prognosis was better for patients in the low-risk group than in the high-risk group (**Fig. 1K** and **M**). Moreover, to validate the value of the CMRPS score in immunotherapy sensitivity, we compared IPS between CMRPS groups, and a higher IPS was observed in the low-risk group than in the high-risk group with the single or the combined treatment of CTLA-4 and PD-1 (*P* < 0.05, **Fig. S2C−F**). This further confirms the distinct immunotherapy responder patients with STAD in different CMRPS groups and shows that a potential relationship exists between copper metabolism and immunotherapy. Therefore, it could be proposed that copper metabolism in STAD is a new target for the immunotherapy of STAD, and CMRPS is a new immunotherapeutic biomarker for STAD.

It has been demonstrated that copper metabolism is associated with a variety of oncology drug treatments. Ishida et al. illustrated that copper chelators increased the level of cisplatin−DNA adducts in cervical tumor tissues and also enhanced the killing effect of cisplatin on cervical tumor cancer cells.^63^ Budman et al. showed that copper-lowering agents increased the therapeutic effect of docetaxel in breast cancer.^64^ Fu et al. demonstrated that the combination of copper-lowering agents with carboplatin was effective and had antitumor activity in epithelial ovarian cancer, head and neck cancer, and non-small cell lung cancer.^65^ We studied the association between CMRPS and the antitumor drug response to facilitate personalized treatment of STAD. It was revealed that the sensitivity of commonly used antitumor drugs (axitinib, cisplatin, oxaliplatin, gemcitabine, and sorafenib) was higher in the low-risk group than the high-risk group of CMRPS, indicating that the CMRPS score could be used to evaluate the benefit and aptness of chemotherapy for patients with STAD, which may contribute to the strategic development of personalized treatment (**Fig. 1O**). In summary, mediating copper metabolism in STAD is a novel direction for future oncology drug therapy.

Moreover, to further explore the potential medications that target 11 DEGs-_CMRPS_ and inhibit copper metabolism, we identified 141 drug and gene pairs based on the CellMiner database (**Appendix F**). After a careful literature survey of the nine top medications (**Fig. S2G**), we noticed that the efficient antitumor effect of cyclophosphamide had been verified in gastric cancer, and its potential in the combination treatment of gastric cancer has been emphasized. AMG-232 is a drug aimed at acute myeloid leukemia.^66^ It enhanced T cell killing of ovarian clear cell carcinoma cell lines and was expected to be a part of the combined immunotherapy.^67^ INK-128 is a medication to treat tumors, such as pancreatic cancer^68^ and osteosarcoma^69^. ABT-199 is a drug designed to treat leukemia.^70^ Imexon is used to treat cancers, including melanoma and multiple myeloma.^71^ GSK-1904529A is adopted for the treatment of solid and hematologic malignancies.^72^ P-529 is reported to treat glioma.^73^ Nandrolone phenpropionate is indicated for the treatment of breast cancer.^74^ These DEGs-_CMRPS_-targeted drugs demonstrated exceptional function in various cancer cells and may have outstanding results in the treatment of gastric adenocarcinoma. However, the detailed effects of DEGs-_CMRPS_-targeted drugs in treating STAD still need further elucidation.

Our results systematically explored the functional mechanism of copper metabolism in STAD and provided new insights into immunotherapy for STAD based on copper metabolism. However, considering the limitations of bioinformatics research, marked by a lack of experimental investigations and clinical verifications, we suggest a careful interpretation of the results in this study and encourage a further study on the relationship among copper metabolism, the tumor microenvironment, and immunotherapy in STAD.

**Conclusion**

In this study, we selected samples from the TCGA database to investigate the prognostic value and immunotherapy sensitivity of a CMRPS in STAD. Our analysis resulted in the identification of 11 DEGs-_CMRPS_, which were associated with patient prognosis, and the discovery of 141 potential drugs targeting these genes. Furthermore, we identified four immune genes and three anti-immune genes among the DEGs-_CMRPS_. Additionally, pathway enrichment analysis by GSVA revealed that copper metabolism might play important roles in immune-related pathways and metabolic reprogramming-related pathways in STAD. Notably, copper metabolism can affect programmed cell death and anticancer drug sensitivity. Overall, our study contributes to the understanding of the relationship between copper metabolism, the tumor microenvironment, and immunotherapy in STAD. These findings provide valuable insights for the identification of novel prognostic markers, therapeutic targets, and indicators for immunotherapeutic strategies in clinical research and the treatment of STAD.

**Supplementary Figures and Tables**

Figure S1. DEGs and DEGs-_CM_ in STAD.

Figure S2. Mutation landscape and immunotherapy response in high- and low-risk groups of the CMRPS model.

Table S1. Eleven DEGs-_CMRPS_ in the CMRPS model in STAD.

Table S2. Comparison of 11 mRNAs expression changes in this study and previous cancer studies.

**Appendix**

Appendix A. One hundred and thirty-eight genes involved in copper metabolism.

Appendix B. Fifty-nine KEGG pathways obtained through GSVA.

Appendix C. Five programmed cell death gene sets.

Appendix D. Twenty-three immune cell infiltration gene sets.

Appendix E. Fifteen immune function gene sets.

Appendix F. Potential drugs targeting 11 DEGs-_CMRPS_.

**References**

1. Sung H, Ferlay J, Siegel RL, et al. Global Cancer Statistics 2020: GLOBOCAN Estimates of Incidence and Mortality Worldwide for 36 Cancers in 185 Countries. *CA Cancer J Clin.* 2021;71(3):209-249.

2. Yang M, Wu X, Hu J, et al. COMMD10 inhibits HIF1alpha/CP loop to enhance ferroptosis and radiosensitivity by disrupting Cu-Fe balance in hepatocellular carcinoma. *J Hepatol.* 2022;76(5):1138-1150.

3. Shanbhag VC, Gudekar N, Jasmer K, Papageorgiou C, Singh K, Petris MJ. Copper metabolism as a unique vulnerability in cancer. *Biochim Biophys Acta Mol Cell Res.* 2021;1868(2):118893.

4. Ge EJ, Bush AI, Casini A, et al. Connecting copper and cancer: from transition metal signalling to metalloplasia. *Nat Rev Cancer.* 2022;22(2):102-113.

5. Li Y. Copper homeostasis: Emerging target for cancer treatment. *IUBMB Life.* 2020;72(9):1900-1908.

6. Ruiz LM, Libedinsky A, Elorza AA. Role of Copper on Mitochondrial Function and Metabolism. *Front Mol Biosci.* 2021;8:711227.

7. Tsvetkov P, Coy S, Petrova B, et al. Copper induces cell death by targeting lipoylated TCA cycle proteins. *Science.* 2022;375(6586):1254-1261.

8. Jiang Y, Huo Z, Qi X, Zuo T, Wu Z. Copper-induced tumor cell death mechanisms and antitumor theragnostic applications of copper complexes. *Nanomedicine (Lond).* 2022;17(5):303-324.

9. Tsang T, Posimo JM, Gudiel AA, Cicchini M, Feldser DM, Brady DC. Copper is an essential regulator of the autophagic kinases ULK1/2 to drive lung adenocarcinoma. *Nat Cell Biol.* 2020;22(4):412-424.

10. Luo Q, Song Y, Kang J, et al. mtROS-mediated Akt/AMPK/mTOR pathway was involved in Copper-induced autophagy and it attenuates Copper-induced apoptosis in RAW264.7 mouse monocytes. *Redox Biol.* 2021;41:101912.

11. Gao W, Huang Z, Duan J, Nice EC, Lin J, Huang C. Elesclomol induces copper-dependent ferroptosis in colorectal cancer cells via degradation of ATP7A. *Mol Oncol.* 2021;15(12):3527-3544.

12. Joshi SS, Badgwell BD. Current treatment and recent progress in gastric cancer. *CA Cancer J Clin.* 2021;71(3):264-279.

13. Kang YK, Boku N, Satoh T, et al. Nivolumab in patients with advanced gastric or gastro-oesophageal junction cancer refractory to, or intolerant of, at least two previous chemotherapy regimens (ONO-4538-12, ATTRACTION-2): a randomised, double-blind, placebo-controlled, phase 3 trial. *Lancet (London, England).* 2017;390(10111):2461-2471.

14. Fuchs CS, Doi T, Jang RW, et al. Safety and Efficacy of Pembrolizumab Monotherapy in Patients With Previously Treated Advanced Gastric and Gastroesophageal Junction Cancer: Phase 2 Clinical KEYNOTE-059 Trial. *JAMA oncology.* 2018;4(5):e180013.

15. Muro K, Chung HC, Shankaran V, et al. Pembrolizumab for patients with PD-L1-positive advanced gastric cancer (KEYNOTE-012): a multicentre, open-label, phase 1b trial. *The Lancet Oncology.* 2016;17(6):717-726.

16. Jett KA, Baker ZN, Hossain A, et al. Mitochondrial dysfunction reactivates alpha-fetoprotein expression that drives copper-dependent immunosuppression in mitochondrial disease models. *J Clin Invest.* 2023;133(1):e154684.

17. Peng L, Peng JY, Cai DK, et al. Immune Infiltration and Clinical Outcome of Super-Enhancer-Associated lncRNAs in Stomach Adenocarcinoma. *Front Oncol.* 2022;12:780493.

18. Zhang X, Wang Y, A G, Qu C, Chen J. Pan-Cancer Analysis of PARP1 Alterations as Biomarkers in the Prediction of Immunotherapeutic Effects and the Association of Its Expression Levels and Immunotherapy Signatures. *Front Immunol.* 2021;12:721030.

19. He C, Zhu X, Kong F, et al. The Value of m5C-Related lncRNAs in the Prognostic Assessment and Immunotherapy of Stomach Adenocarcinoma. *Biomed Res Int.* 2022;2022:2747799.

20. Liberzon A, Subramanian A, Pinchback R, Thorvaldsdottir H, Tamayo P, Mesirov JP. Molecular signatures database (MSigDB) 3.0. *Bioinformatics.* 2011;27(12):1739-1740.

21. Liberzon A, Birger C, Thorvaldsdottir H, Ghandi M, Mesirov JP, Tamayo P. The Molecular Signatures Database (MSigDB) hallmark gene set collection. *Cell Syst.* 2015;1(6):417-425.

22. Zhou N, Bao J. FerrDb: a manually curated resource for regulators and markers of ferroptosis and ferroptosis-disease associations. *Database (Oxford).* 2020;2020:baaa021.

23. Charoentong P, Finotello F, Angelova M, et al. Pan-cancer Immunogenomic Analyses Reveal Genotype-Immunophenotype Relationships and Predictors of Response to Checkpoint Blockade. *Cell Rep.* 2017;18(1):248-262.

24. He Y, Jiang Z, Chen C, Wang X. Classification of triple-negative breast cancers based on Immunogenomic profiling. *J Exp Clin Cancer Res.* 2018;37(1):327.

25. Goldman MJ, Craft B, Hastie M, et al. Visualizing and interpreting cancer genomics data via the Xena platform. *Nat Biotechnol.* 2020;38(6):675-678.

26. Mayakonda A, Lin DC, Assenov Y, Plass C, Koeffler HP. Maftools: efficient and comprehensive analysis of somatic variants in cancer. *Genome Res.* 2018;28(11):1747-1756.

27. Mezquita L, Preeshagul I, Auclin E, et al. Predicting immunotherapy outcomes under therapy in patients with advanced NSCLC using dNLR and its early dynamics. *Eur J Cancer.* 2021;151:211-220.

28. Jardim DL, Goodman A, de Melo Gagliato D, Kurzrock R. The Challenges of Tumor Mutational Burden as an Immunotherapy Biomarker. *Cancer Cell.* 2021;39(2):154-173.

29. Jiang P, Gu S, Pan D, et al. Signatures of T cell dysfunction and exclusion predict cancer immunotherapy response. *Nat Med.* 2018;24(10):1550-1558.

30. Thibodeau SN, Bren G, Schaid D. Microsatellite instability in cancer of the proximal colon. *Science.* 1993;260(5109):816-819.

31. Yang W, Soares J, Greninger P, et al. Genomics of Drug Sensitivity in Cancer (GDSC): a resource for therapeutic biomarker discovery in cancer cells. *Nucleic Acids Res.* 2013;41(Database issue):D955-961.

32. Wu Q, Qian W, Sun X, Jiang S. Small-molecule inhibitors, immune checkpoint inhibitors, and more: FDA-approved novel therapeutic drugs for solid tumors from 1991 to 2021. *J Hematol Oncol.* 2022;15(1):143.

33. Shankavaram UT, Varma S, Kane D, et al. CellMiner: a relational database and query tool for the NCI-60 cancer cell lines. *BMC Genomics.* 2009;10:277.

34. Reinhold WC, Sunshine M, Liu H, et al. CellMiner: a web-based suite of genomic and pharmacologic tools to explore transcript and drug patterns in the NCI-60 cell line set. *Cancer Res.* 2012;72(14):3499-3511.

35. Denoyer D, Masaldan S, La Fontaine S, Cater MA. Targeting copper in cancer therapy: 'Copper That Cancer'. *Metallomics.* 2015;7(11):1459-1476.

36. Lun X, Wells JC, Grinshtein N, et al. Disulfiram when Combined with Copper Enhances the Therapeutic Effects of Temozolomide for the Treatment of Glioblastoma. *Clin Cancer Res.* 2016;22(15):3860-3875.

37. Parmar A, Pascali G, Voli F, et al. In vivo [(64)Cu]CuCl(2) PET imaging reveals activity of Dextran-Catechin on tumor copper homeostasis. *Theranostics.* 2018;8(20):5645-5659.

38. Cheng F, Peng G, Lu Y, et al. Relationship between copper and immunity: The potential role of copper in tumor immunity. *Front Oncol.* 2022;12:1019153.

39. Han YL, Chen L, Qin R, Wang GQ, Lin XH, Dai GH. Lysyl oxidase and hypoxia-inducible factor 1alpha: biomarkers of gastric cancer. *World J Gastroenterol.* 2019;25(15):1828-1839.

40. Guan Y, Xu B, Sui Y, et al. Pan-Cancer Analysis and Validation Reveals that D-Dimer-Related Genes are Prognostic and Downregulate CD8(+) T Cells via TGF-Beta Signaling in Gastric Cancer. *Front Mol Biosci.* 2022;9:790706.

41. Wang J, Zhou Y, Fei X, et al. ADAM9 functions as a promoter of gastric cancer growth which is negatively and post-transcriptionally regulated by miR-126. *Oncol Rep.* 2017;37(4):2033-2040.

42. Wang YY, Ye ZY, Li L, Zhao ZS, Shao QS, Tao HQ. ADAM 10 is associated with gastric cancer progression and prognosis of patients. *J Surg Oncol.* 2011;103(2):116-123.

43. Ye Y, Li X, Yang J, et al. MDM2 is a useful prognostic biomarker for resectable gastric cancer. *Cancer Sci.* 2013;104(5):590-598.

44. Shen X, Wang M, Chen W, et al. Senescence-related genes define prognosis, immune contexture, and pharmacological response in gastric cancer. *Aging (Albany NY).* 2023;15(8):2891-2905.

45. Li D, Zeng Z, Yu T, et al. Expression and clinical implication of S100A12 in gastric carcinoma. *Tumour Biol.* 2016;37(5):6551-6559.

46. Xu G, Fan L, Zhao S, OuYang C. MT1G inhibits the growth and epithelial-mesenchymal transition of gastric cancer cells by regulating the PI3K/AKT signaling pathway. *Genet Mol Biol.* 2022;45(1):e20210067.

47. Hung KC, Huang TC, Cheng CH, et al. The Expression Profile and Prognostic Significance of Metallothionein Genes in Colorectal Cancer. *Int J Mol Sci.* 2019;20(16):3849.

48. Hancq S, Salmon I, Brotchi J, et al. S100A5: a marker of recurrence in WHO grade I meningiomas. *Neuropathol Appl Neurobiol.* 2004;30(2):178-187.

49. Xu M, Wang X, Li Y, et al. Arachidonic Acid Metabolism Controls Macrophage Alternative Activation Through Regulating Oxidative Phosphorylation in PPARgamma Dependent Manner. *Front Immunol.* 2021;12:618501.

50. McFadyen MC, McLeod HL, Jackson FC, Melvin WT, Doehmer J, Murray GI. Cytochrome P450 CYP1B1 protein expression: a novel mechanism of anticancer drug resistance. *Biochem Pharmacol.* 2001;62(2):207-212.

51. Gu J, Huang C, Hu X, Xia J, Shao W, Lin D. Nuclear magnetic resonance-based tissue metabolomic analysis clarifies molecular mechanisms of gastric carcinogenesis. *Cancer Sci.* 2020;111(9):3195-3209.

52. Cui L, Gouw AM, LaGory EL, et al. Mitochondrial copper depletion suppresses triple-negative breast cancer in mice. *Nat Biotechnol.* 2021;39(3):357-367.

53. Blades B, Ayton S, Hung YH, Bush AI, La Fontaine S. Copper and lipid metabolism: A reciprocal relationship. *Biochim Biophys Acta Gen Subj.* 2021;1865(11):129979.

54. Liu X, Peng G. Mitochondria orchestrate T cell fate and function. *Nat Immunol.* 2021;22(3):276-278.

55. Steinert EM, Vasan K, Chandel NS. Mitochondrial Metabolism Regulation of T Cell-Mediated Immunity. *Annu Rev Immunol.* 2021;39:395-416.

56. Liu Y, Zhou Z, Hou J, et al. Tumor Selective Metabolic Reprogramming as a Prospective PD-L1 Depression Strategy to Reactivate Immunotherapy. *Adv Mater.* 2022;34(41):e2206121.

57. Liao Y, Zhao J, Bulek K, et al. Inflammation mobilizes copper metabolism to promote colon tumorigenesis via an IL-17-STEAP4-XIAP axis. *Nat Commun.* 2020;11(1):900.

58. Carvalho A, Lu J, Francis JD, et al. S100A12 in Digestive Diseases and Health: A Scoping Review. *Gastroenterol Res Pract.* 2020;2020:2868373.

59. Zhou J, Kryczek I, Li S, et al. The ubiquitin ligase MDM2 sustains STAT5 stability to control T cell-mediated antitumor immunity. *Nat Immunol.* 2021;22(4):460-470.

60. Huang X, Wang L, Guo H, Zhang W, Shao Z. Single-cell transcriptomics reveals the regulative roles of cancer associated fibroblasts in tumor immune microenvironment of recurrent osteosarcoma. *Theranostics.* 2022;12(13):5877-5887.

61. Umeda M, Yoshida N, Hisada R, et al. ADAM9 enhances Th17 cell differentiation and autoimmunity by activating TGF-beta1. *Proc Natl Acad Sci U S A.* 2021;118(18):e2023230118.

62. Wang J, Kotagiri P, Lyons PA, et al. Coagulation factor V is a T-cell inhibitor expressed by leukocytes in COVID-19. *iScience.* 2022;25(3):103971.

63. Ishida S, McCormick F, Smith-McCune K, Hanahan D. Enhancing tumor-specific uptake of the anticancer drug cisplatin with a copper chelator. *Cancer Cell.* 2010;17(6):574-583.

64. Budman DR, Calabro A. In vitro search for synergy and antagonism: evaluation of docetaxel combinations in breast cancer cell lines. *Breast Cancer Res Treat.* 2002;74(1):41-46.

65. Fu S, Hou MM, Wheler J, et al. Exploratory study of carboplatin plus the copper-lowering agent trientine in patients with advanced malignancies. *Invest New Drugs.* 2014;32(3):465-472.

66. Erba HP, Becker PS, Shami PJ, et al. Phase 1b study of the MDM2 inhibitor AMG 232 with or without trametinib in relapsed/refractory acute myeloid leukemia. *Blood Adv.* 2019;3(13):1939-1949.

67. Sahin I, Zhang S, Navaraj A, et al. AMG-232 sensitizes high MDM2-expressing tumor cells to T-cell-mediated killing. *Cell Death Discov.* 2020;6:57.

68. Lou HZ, Weng XC, Pan HM, et al. The novel mTORC1/2 dual inhibitor INK-128 suppresses survival and proliferation of primary and transformed human pancreatic cancer cells. *Biochem Biophys Res Commun.* 2014;450(2):973-978.

69. Jiang H, Zeng Z. Dual mTORC1/2 inhibition by INK-128 results in antitumor activity in preclinical models of osteosarcoma. *Biochem Biophys Res Commun.* 2015;468(1-2):255-261.

70. Souers AJ, Leverson JD, Boghaert ER, et al. ABT-199, a potent and selective BCL-2 inhibitor, achieves antitumor activity while sparing platelets. *Nature medicine.* 2013;19(2):202-208.

71. Scott J, Dorr RT, Samulitis B, Landowski TH. Imexon-based combination chemotherapy in A375 human melanoma and RPMI 8226 human myeloma cell lines. *Cancer Chemother Pharmacol.* 2007;59(6):749-757.

72. Sabbatini P, Rowand JL, Groy A, et al. Antitumor activity of GSK1904529A, a small-molecule inhibitor of the insulin-like growth factor-I receptor tyrosine kinase. *Clin Cancer Res.* 2009;15(9):3058-3067.

73. Xue Q, Hopkins B, Perruzzi C, Udayakumar D, Sherris D, Benjamin LE. Palomid 529, a novel small-molecule drug, is a TORC1/TORC2 inhibitor that reduces tumor growth, tumor angiogenesis, and vascular permeability. *Cancer Res.* 2008;68(22):9551-9557.

74. Sirianni R, Capparelli C, Chimento A, et al. Nandrolone and stanozolol upregulate aromatase expression and further increase IGF-I-dependent effects on MCF-7 breast cancer cell proliferation. *Mol Cell Endocrinol.* 2012;363(1-2):100-110.

**
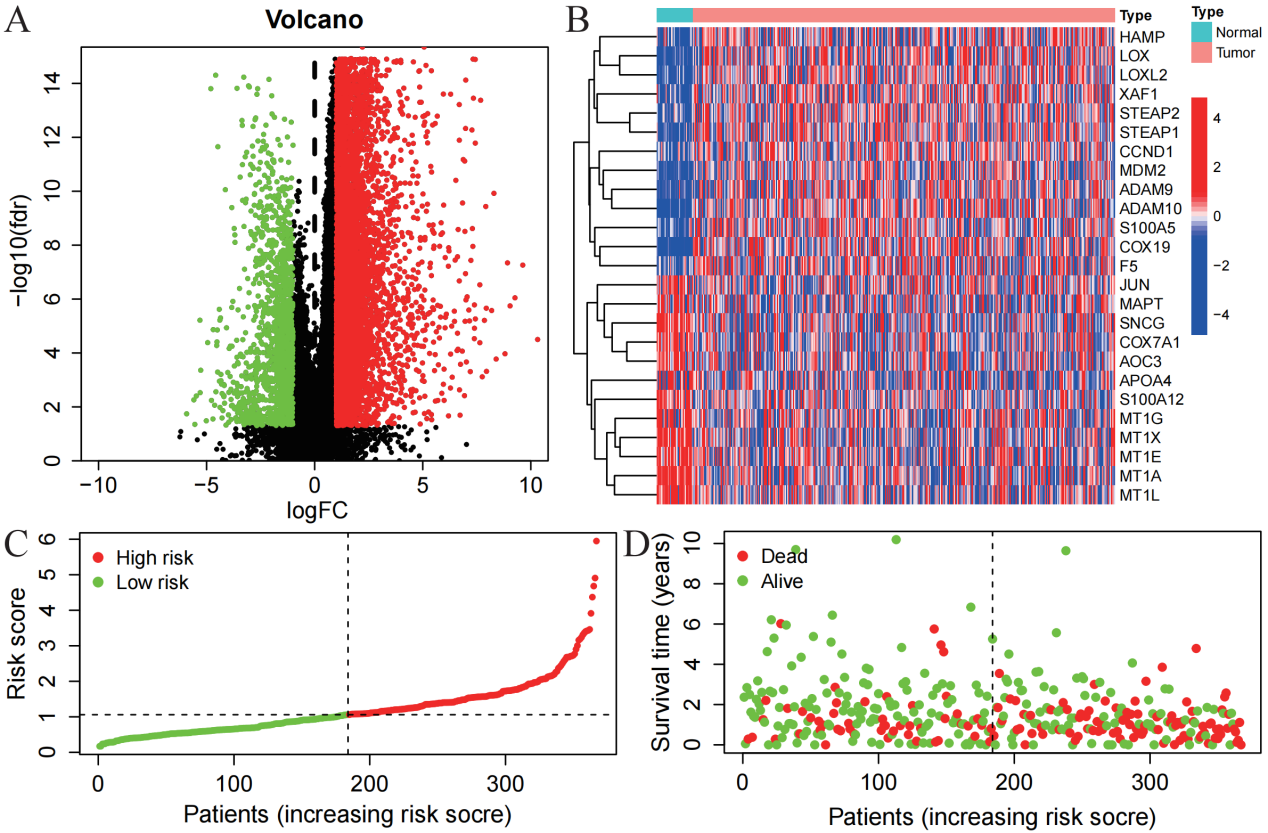
 Figure S1.** DEGs and DEGs_-CM_ in STAD. (**A**) Volcano plot of DEGs; red dots represent upregulation, and green dots represent downregulation. (**B**) Heatmap of DEGs_-CM_; red represents upregulation, and blue represents downregulation. (**C**) CMRPS score curve; green dots represent low-risk patients, and red dots represent high-risk patients. (**D**) Survival status diagram; green dots denote a live person, and red dots denote a dead person.


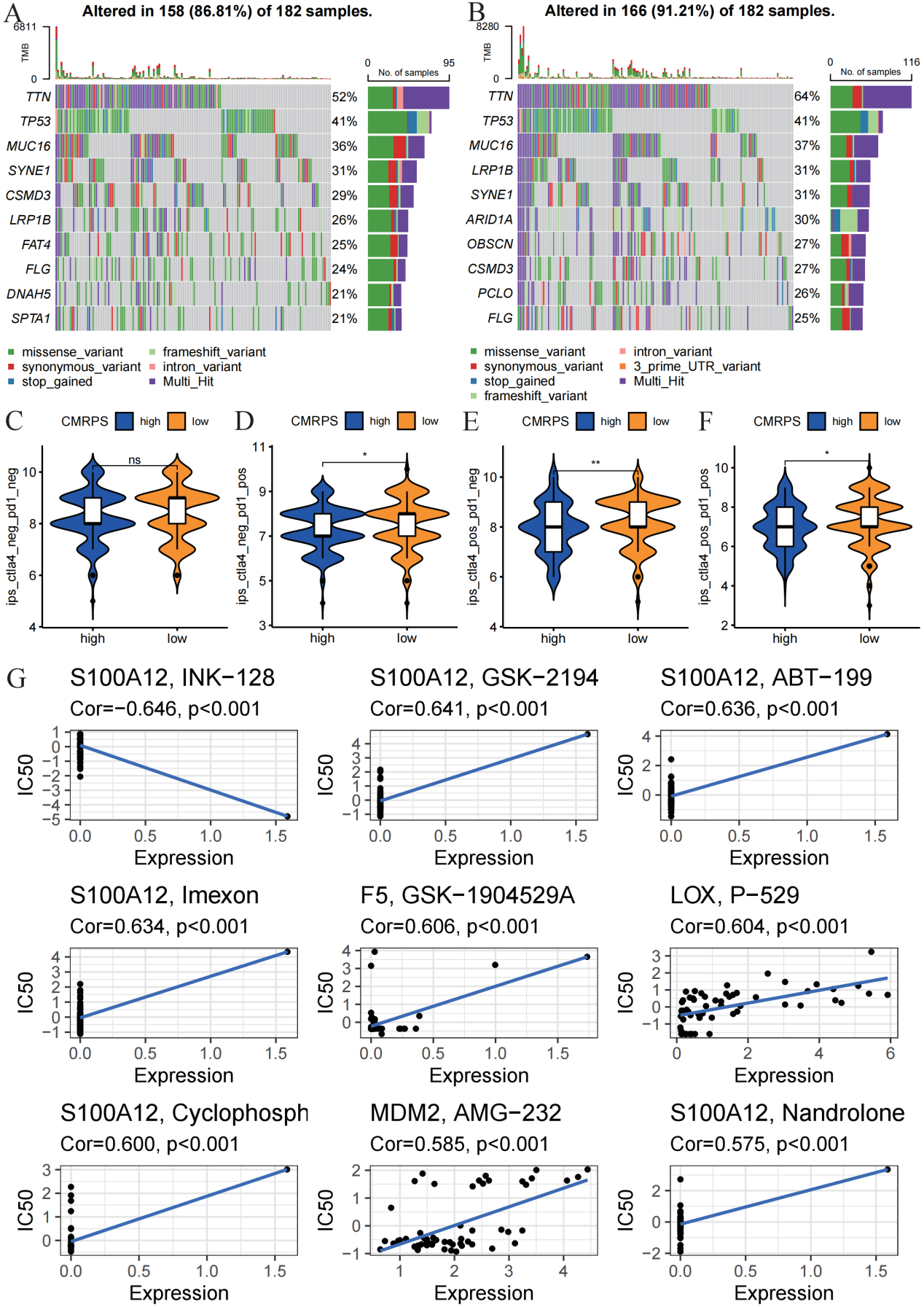


**Figure S2.** Mutation landscape and immunotherapy response in high- and low-risk groups of the CMRPS model. (**A**) The top 10 genes mutation landscape in the high-risk group. (**B**) The top 10 genes mutation landscape in the low-risk group. (**C−F**) Comparison of IPS in two risk groups. (**G**) The abscissa denotes the gene expression level, and the ordinate denotes the half-maximal inhibitory concentration (IC_50_) of medication. Correlation coefficient (*r*). *: *P* < 0.05, **: *P* < 0.01, ns: not significant.

**Table S1.** Eleven DEGs-_CMRPS_ in the CMRPS model in STAD.

| **Expression** | **Gene** | **logFC** | **Coefficient** | **HR** | **HR.95L** | **HR.95H** | ***P*-value** |
| --- | --- | --- | --- | --- | --- | --- | --- |
| Upregulated | LOX | 1.82163 | 0.360331 | 1.433803 | 1.201448 | 1.711095 | 6.48E-05 |
|  | F5 | 2.42327 | 0.178789 | 1.195768 | 1.063459 | 1.344539 | 0.002805 |
|  | S100A5 | 1.55507 | -0.468250 | 0.626096 | 0.430682 | 0.910175 | 0.014166 |
|  | ADAM9 | 1.05336 | 0.268576 | 1.308101 | 1.035637 | 1.652247 | 0.024208 |
|  | ADAM10 | 1.15602 | -0.395240 | 0.673521 | 0.437864 | 1.036007 | 0.072026 |
|  | COX19 | 1.18530 | 0.308465 | 1.361334 | 0.918943 | 2.016697 | 0.123954 |
|  | MDM2 | 1.34047 | -0.178480 | 0.836541 | 0.661449 | 1.057981 | 0.136338 |
| Downregulated | MT1L | -1.05668 | -0.275790 | 0.758975 | 0.609579 | 0.944984 | 0.013666 |
|  | SNCG | -1.09836 | 0.180607 | 1.197944 | 1.030195 | 1.393009 | 0.018953 |
|  | S100A12 | -1.39264 | 0.147769 | 1.159246 | 1.020124 | 1.317339 | 0.023487 |
|  | MT1G | -1.45367 | 0.096311 | 1.101102 | 0.998707 | 1.213995 | 0.053116 |

**Table S2.** Comparison of 11 mRNAs expression changes in this study and previous cancer studies.

| **mRNA** | **Feature** | **Summary** |
| --- | --- | --- |
| *LOX* |  | Han et al.^39^ performed immunohistochemical staining on gastric cancer (GC) tissues and qRT-PCR analysis on GC cells. Overexpression of gene *LOX* was observed in tumor tissues and tumor cells. |
| *F5* |  | Guan et al.^40^ conducted qRT-PCR on STAD tissues and adjacent normal gastric tissues. Results showed higher expression of mRNA *F5* in GC samples compared to the normal samples. |
| *ADAM9* |  | Wang et al.^41^ conducted Western blot and qRT-PCR analysis on GC cell lines. The mRNA level of *ADAM9* was upregulated in GC SGC-7901 cells compared to GES-1 cells. In addition, ADAM9 acted as an initiating factor in regulating GC cell proliferation. |
| *ADAM10* |  | Wang et al.^42^ undertook immunohistochemical detection of STAD samples. The protein level of ADAM10 was increased in tumor tissues compared to adjacent non-cancerous tissues, which positively connected with the infiltration depth, lymph nodes metastasis, distant metastasis, and TNM stage of GC. |
| *MDM2* |  | Ye et al.^43^ performed immunohistochemical detection on STAD samples. The expression levels of protein MDM2 were significantly upregulated in tumor tissue than in adjacent non-cancerous tissue. |
| *SNCG* |  | Shen et al.^44^ carried out immunohistochemical staining and scRNA-seq analysis of SNCG. *SNCG* mRNA was downregulated in tumor tissues compared to non-neoplastic adjacent gastric tissues. |
| *S100A12* |  | Li et al.^45^ carried out immunohistochemical staining on GC tissues. The protein level of S100A12 was lower in cancer tissues than in non-cancerous mucosa tissues. |
| *MT1G* |  | Xu et al.^46^ conducted RT-qPCR and Western blot analysis on STAD cell lines. The expression level of *MT1G* was significantly decreased in tumor cell lines (NCI-N87, SNU-1, HS-746T, and HGC-27) than in a normal gastric cell line (GES-1). |
| *MT1L* |  | Hung et al.^47^ compared colorectal cancer tissues and their adjacent non-tumor tissues. *MT1L* mRNA was downregulated in about 90% (27/30) of colorectal cancer tumor tissues. |
| *S100A5* |  | The relative downregulation of S100A5 in cancer compared with normal samples has not been reported by any experiments before. However, Hancq et al.^48^ performed a histochemical analysis on the fixed tumor material from meningiomas. They elucidated that the expression level of S100A5 was lower in the recurrent cases compared with the non-recurrent cases with complete resection of WHO grade I meningiomas. |
| *COX19* |  | The upregulation of *COX19* has not been reported by any experiments before, and it is a novel tumor biomarker reported for the first time in this study. We found that *COX19* was a copper metabolism-related gene and a member in a Cox risk model relevant to the prognosis of patients with STAD. |

mRNAs upregulated in STAD in accordance with our calculated result.

mRNAs downregulated in STAD in accordance with our calculated result.

mRNAs downregulated in other cancers in accordance with our calculated result.

mRNAs upregulated in our calculated results that have not been verified in cancers.
